# The Influence of Aortic Valve Disease on Coronary Hemodynamics: A Computational Model-Based Study

**DOI:** 10.3390/bioengineering10060709

**Published:** 2023-06-11

**Authors:** Xuanyu Li, Sergey Simakov, Youjun Liu, Taiwei Liu, Yue Wang, Fuyou Liang

**Affiliations:** 1 Department of Engineering Mechanics, School of Naval Architecture, Ocean and Civil Engineering, Shanghai Jiao Tong University, Shanghai 200240, China; lizuanyu@sjtu.edu.cn (X.L.); taiweiliu@sjtu.edu.cn (T.L.); 2 Marchuk Institute of Numerical Mathematics of the Russian Academy of Sciences, Moscow 119991, Russia; simakov.ss@mipt.ru; 3 College of Life Science and Bioengineering, Beijing University of Technology, Beijing 100124, China; lyjlma@bjut.edu.cn; 4 Department of Cardiology, Shanghai Ninth People’s Hospital, Shanghai Jiao Tong University School of Medicine, Shanghai 200011, China; 5 State Key Laboratory of Ocean Engineering, School of Naval Architecture, Ocean and Civil Engineering, Shanghai Jiao Tong University, Shanghai 200240, China

**Keywords:** aortic valve disease, coronary artery disease, multi-scale modeling, hemodynamics

## Abstract

Aortic valve disease (AVD) often coexists with coronary artery disease (CAD), but whether and how the two diseases are correlated remains poorly understood. In this study, a zero–three dimensional (0-3D) multi-scale modeling method was developed to integrate coronary artery hemodynamics, aortic valve dynamics, coronary flow autoregulation mechanism, and systemic hemodynamics into a unique model system, thereby yielding a mathematical tool for quantifying the influences of aortic valve stenosis (AS) and aortic valve regurgitation (AR) on hemodynamics in large coronary arteries. The model was applied to simulate blood flows in six patient-specific left anterior descending coronary arteries (LADs) under various aortic valve conditions (i.e., control (free of AVD), AS, and AR). Obtained results showed that the space-averaged oscillatory shear index (SA-OSI) was significantly higher under the AS condition but lower under the AR condition in comparison with the control condition. Relatively, the overall magnitude of wall shear stress was less affected by AVD. Further data analysis revealed that AS induced the increase in OSI in LADs mainly through its role in augmenting the low-frequency components of coronary flow waveform. These findings imply that AS might increase the risk or progression of CAD by deteriorating the hemodynamic environment in coronary arteries.

## 1. Introduction

Coronary artery disease (CAD) is a major cause of cardiovascular morbidity and mortality worldwide and shares similar risk factors with aortic valve disease (AVD) [1]. Clinical studies revealed that the prevalence of CAD was high in patients with AVD [2,3,4]. For instance, approximately 50% of patients with severe aortic valve stenosis suffered from concomitant CAD [5]. Physiologically, resting blood flow rates in coronary arteries increase with the severity of AVD [6,7] as a consequence of compensatory responses to increased cardiac workload and myocardial oxygen consumption [8,9]. In addition, blood flow velocity waveforms in coronary arteries are also altered by AVD, which usually exhibits a higher diastolic peak in patients with aortic valve stenosis (AS) [10], while a higher systolic peak in patients with aortic valve regurgitation (AR) [11]. Theoretically, AVD-induced changes in both the magnitude and waveform of coronary blood flow would affect hemodynamic characteristics in coronary arteries. So far, how AVD would alter coronary hemodynamic characteristics and whether the alterations could be associated with CAD remain poorly understood.

Hemodynamic metrics such as wall shear stress (WSS) and its derivatives have been extensively proven to be related to the initiation and development of atherosclerotic lesions [12]. For instance, low time-averaged WSS (TAWSS) and high oscillatory shear index (OSI) in coronary arteries were found to be closely associated with the progression of CAD through their roles in inducing endothelial cell damage and triggering inflammatory reactions [13,14]. Existing biomechanical studies on AVD were generally focused on the changes in flow patterns in the vicinity of aortic valves [15,16,17]. Some studies addressed the influences of the dynamic motion of aortic valve leaflets on blood flows in the proximal segments of coronary arteries [18,19,20], finding that AS significantly reduces flow velocity and WSS at the entrance of coronary arteries, which might increase the risk of coronary atherosclerosis. Most of these studies were, however, based on idealized models and did not take into account AVD-induced changes in coronary flow velocity waveform. In addition, models developed in these studies did not incorporate the autoregulatory mechanism of coronary blood flow, which, under in vivo conditions, is critical for regulating blood flow to meet the increased myocardial perfusion demand in the presence of AVD [21,22,23]. These limitations rendered the studies unable to fully elucidate the impacts of AVD on hemodynamic characteristics in coronary arteries.

The main aim of this study was to clarify how AS and AR would alter blood flow patterns and major hemodynamic metrics (e.g., TAWSS, OSI) in coronary arteries under physiological conditions. For any three-dimensional (3D) models of local artery segments, the definition of inflow and outflow boundary conditions (BCs) is mandatory. Previous studies have demonstrated that many hemodynamic quantities, especially WSS metrics, are sensitive to the characteristics of BCs, such as the magnitude and shape of the flow waveform imposed at the inlet, prescribed flow velocity profile, and the parameters of boundary models [24,25,26,27,28], suggesting that correct setting of BCs in localized 3D modeling is an important factor determining the physiological fidelity of the numerical outcome. For our study, how to incorporate the influence of AVD into the modeling and computation of coronary artery blood flow is a critical issue. At this point, geometrical multi-scale modeling may be a practical approach, which has been widely applied in previous studies to account for the interactions between systemic hemodynamics and 3D blood flows in local vascular regions of interest [29,30,31]. Following the concept, we developed a zero–three dimensional (0-3D) multi-scale modeling method to integrate coronary artery hemodynamics, aortic valve dynamics, coronary flow autoregulation mechanism, and systemic hemodynamics into a unique model system. In the model system, the coronary artery segment of interest is represented by a 3D model coupled to 0D models of other cardiovascular portions. In this way, the BCs of the 3D model are autonomously generated via coupled computation of hemodynamic variables at the 0D/3D model interfaces, which can also respond spontaneously to changes in pathophysiological conditions, such as cardiac valve disease, artery stenosis, and vasodilation of distal vessels. In this study, patient-specific coronary artery models reconstructed from medical images were incorporated into the model system and tested with respect to hemodynamic changes upon the introduction of various AVDs.

## 2. Materials and Methods

**Figure 1:**
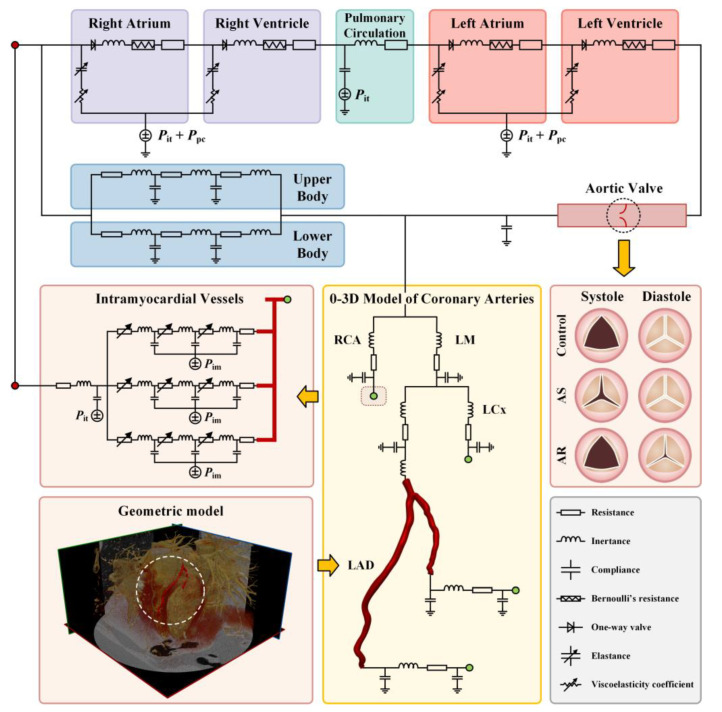
Schematic description of 0-3D multi-scale modeling of coronary hemodynamics. The left lower panel shows CCTA image-based reconstruction of a 3D geometric model for a LAD; the middle lower panel displays the connections between the 3D model of a LAD with the upstream and downstream 0D models; the right middle panel illustrates the opening state of aortic valve during systole and diastole under normal and various pathological conditions; and other panels show the configuration of 0D models.

The 0-3D multi-scale model consisted of a 3D model of a coronary artery segment, a 0D (or lumped parameter) model of the coronary circulation, and a 0D model of the systemic circulation (including the aortic valve) (see Figure 1). In the multi-scale model, the 3D model is used to simulate the detailed flow patterns and quantify hemodynamic variables in a coronary artery segment of interest. Additionally, the 0D models are responsible for providing physiologically reasonable boundary conditions for the 3D model while taking into account the influences of aortic valve disease, coronary flow autoregulation, and other cardiovascular factors on coronary blood flow waveform.

Multi-scale models were built for six coronary arteries free of or with stenosis, and blood flow in each coronary artery was simulated under three aortic valve conditions (i.e., normal, AS, and AR). As a consequence, eighteen cases of simulations were carried out. 

### 2.1. Reconstruction of Geometric Models of Coronary Arteries Based on Medical Images and Mesh Generation

We collected clinical data from six patients who were suspected of CAD and underwent CCTA (coronary computed tomographic angiography) scanning in the Shanghai Ninth People’s Hospital (Shanghai, China). This study was part of clinical research on CAD, which has been approved by the ethics committee of the hospital. The 3D geometric model of the left anterior descending coronary artery (LAD) was reconstructed from the CCTA images of each patient via automatic threshold segmentation, manual adjustment, and smoothing using Mimics (ver. 16.0, Materialise, Leuven, Belgium). Based on the reconstructed models, the severity of stenosis in LAD was assessed by diameter stenosis (DS), and the six LADs were allocated to three groups, namely the stenosis-free (DS ≈ 0%) group, the mild stenosis (DS < 25%) group, and the severe stenosis (DS > 75%) group, with each group containing two LADs. Straight extension tubes (with lengths set to 15 times the inlet or outlet diameter) were further added to the inlet and outlet of each geometric model, which was expected to play a role in minimizing the influences on the simulation of blood flows in arterial segments of interest from artifacts introduced by the prescription of inflow/outflow boundary conditions (e.g., fixed cross-sectional flow velocity profile at inlet, and uniform in-plane distribution of pressure at outlet) [32,33].

Each geometric model was imported into ICEM CFD (ANSYS Inc., Canonsburg, PA, USA) to generate a mesh model that would be used to discretize and numerically solve the governing equations of blood flow. Herein, a hybrid meshing strategy was adopted, where the center space of lumen is divided by tetrahedral elements, and the region adjacent to the wall is divided by prism elements which form five layers of structured meshes that will help to improve the accuracy of hemodynamic computation in the near-wall zones where flow velocity gradients are large. Grid sensitivity analysis was conducted on a selected model to determine the appropriate element size. Herein, we fixed the thickness of the first prism element layer (the thinnest layer adjacent to the wall) at 0.012 mm while reducing the maximum size of tetrahedral elements from 0.55 mm to 0.25 mm at an interval of 0.1 mm to generate a series of mesh models, and, accordingly, carried out hemodynamic simulations with the same boundary conditions. The outcome showed that the hemodynamic parameters of interest (i.e., space-averaged TAWSS and OSI) changed by less than 0.5% when the element size was reduced from 0.35 mm to 0.25 mm, which indicates that a maximum size of 0.25 mm for the tetrahedral elements is sufficient to achieve mesh-independent numerical solutions. The maximum size of tetrahedral elements and the thickness of the first prism element layer was applied to all the models, and the total number of elements contained in each mesh model varied from 1.52 million to 1.71 million depending on the complexity and size of the geometric model.

### 2.2. Development of the 0-3D Multi-Scale Model

#### 2.2.1. Model Configuration

In the 0-3D multi-scale model, the 3D model is a LAD model reconstructed from medical images (as described above), with its inlet being connected to 0D models of the proximal coronary arteries and the systemic cardiovascular system, and its outlets supported by 0D models of distal coronary vessels (see Figure 1). More specifically, the 0D model of the cardiovascular system was adapted from a model developed in a previous study of our group [34], which was established based on simplified compartmentalization of the system into cardiac chambers, pulmonary circulation, and systemic circulation (divided into upper body and lower body parts). Each compartment was modeled using lumped parameters representing the specific properties of the components inside (e.g., viscous resistance (R) and compliance (C) of vessels, inertance of blood (L), elastance (E) of cardiac chamber, and Bernoulli’s resistance (B) of cardiac valve). 

A similar methodology was employed to build a 0D model for the coronary circulation, except for the need to construct multi-layer models for intramyocardial vessels to account for the hemodynamic effects of intramyocardial pressure (*P_im_*) that changes periodically with heart beating and varies spatially in the myocardium. Herein, the three-layer modeling method developed in a previous study of our group [35] was adopted. In brief, the intramyocardial vessels distal to each large epicardial coronary artery were divided into three layers located in the subepicardium, mid-wall, and subendocardium, respectively (see the left middle panel of Figure 1). The outer walls of vessels in the three myocardial layers were exposed to different intramyocardial pressures that increase linearly from the subepicardium to the subendocardium. In addition, the resistances of intramyocardial microvessels (specifically, small arteries and arterioles) can be modulated to incorporate the coronary flow autoregulation mechanism.

#### 2.2.2. Governing Equations and Numerical Methods

Blood flow in the 3D model of LAD was treated as an incompressible viscous Newtonian fluid governed by the unsteady continuity and Navier–Stokes equations. The density and dynamic viscosity of blood were set at 1050 kg/m^3^ and 0.0035 Pa s, respectively. The deformation of coronary artery wall was ignored in 3D modeling by adopting the rigid-wall assumption in order to avoid solving the time-consuming fluid–structure interaction problem that would greatly increase the computational cost of the multi-scale model where the 3D model needs to be solved repeatedly for many cardiac cycles to reach numerical convergence of the entire model system. The simplification is acceptable since this present study is concerned with time-averaged WSS metrics in coronary arteries, which have been proved by previous studies [36,37] to be only slightly affected by wall deformation. The inflow and outflow boundary conditions were determined automatically by the coupling between the 3D and 0D models (will be detailed later). The governing equations of blood flow were discretized on the mesh model and numerically solved using a finite volume method-based CFD software package, FLUENT (ANSYS Inc., Canonsburg, PA, USA). 

The governing equations of the 0D models are ordinary differential ones that were solved using the explicit fourth-order Runge–Kutta method [38]. For more details on the governing equations and numerical methods for the 0D models, please refer to our previous studies [34,39,40].

#### 2.2.3. Numerical Algorithm for Coupling the 3D Model to the 0D Models

**Figure 2:**
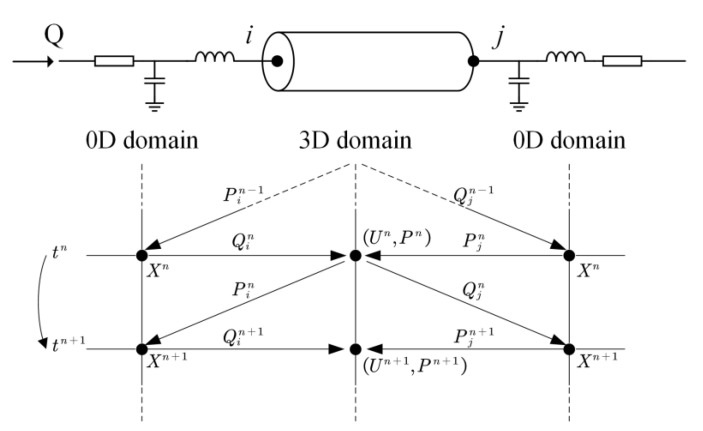
Schematic diagram of data exchanges between 3D and 0D models at the boundaries of 3D model. *t^n^* (=*n*∆*t*) and *t^n^*^+1^ (=(*n* + 1)∆*t*) indicate the current and next time steps; *P*, *Q*, and *U* stand for blood pressure, flow rate, and flow velocity, respectively; and ‘*i*’ and ‘*j*’ denote the inlet and outlet of the 3D model. The data exchanges proceed in a time-marking manner.

Given the fact that the 3D and 0D models are governed by different equation systems that cannot be solved simultaneously with a single solver, we developed a numerical coupling algorithm to colligate the solutions of the two types of models at each numerical time step. The algorithm made use of the benefit of the explicit Runge–Kutta method for solving the 0D models, i.e., given the solution and boundary conditions at current time step, the numerical solution at the next time step can be obtained explicitly. The flowchart of the algorithm is as follows: Step 1: at time step t=nΔt, use the solutions of the 0D models to set the inlet flow rate and outlet pressure of the 3D model; Step 2: solve the 3D model and pass the computed inlet pressure and outlet flow rate back to the 0D models as boundary conditions to solve for the boundary conditions of the 3D model at the next time step; Step 3: continue the 0D–3D coupling in a time-marching manner for several cardiac cycles until a periodic solution is obtained (see Figure 2).

**Figure 3:**
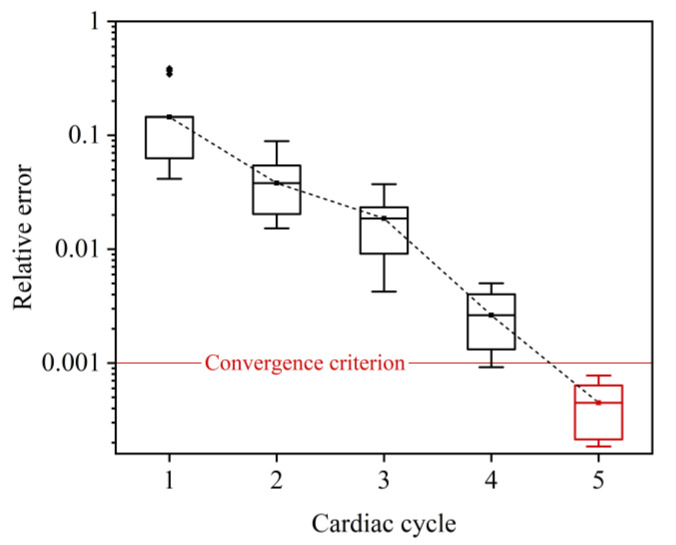
Relative errors of 3D model-computed flow rate waveforms in two consecutive cardiac cycles during time-marching 0-3D simulations. Since the errors differ slightly between models, the errors for all six LAD models are statistically illustrated in form of box plot. The convergence criterion was set as <0.001, which was reached after the 0-3D simulations ran for five cardiac cycles.

The coupling algorithm and the imposition of boundary conditions were implemented using embedding coded UDFs (User Defined Functions) in FLUENT. The time steps of the 3D and 0D models were both fixed at 0.001 s, and the global Courant number was lower than 0.8. Convergence of the numerical coupling was judged when the relative errors of the computed mean flow rates by the 3D and 0D models in two consecutive cardiac cycles were both less than 0.1%. Figure 3 shows that the numerical coupling converged within five cardiac cycles for all six LAD models. Each set of numerical simulations was run on a workstation equipped with an AMD EPYC 7542 32-Core Processor and 256 GB DDR RAM and was completed in about 48 h.

#### 2.2.4. Modeling of Aortic Valve Disease and Coronary Flow Autoregulation

In this study, we modified the lumped-parameter valve model adopted in previous studies [34,38] to represent trans-valvular hemodynamics under normal and pathological conditions [35]. In the model, the pressure drop ($\Delta P_{av}$) across the aortic valve was related to the trans-valvular flow rate ($Q_{av}$) as follows:

(1) $$\Delta P_{av}=R_{av}Q_{av}+B_{av}Q_{av}\left|Q_{av}\right|+L_{av}\frac{dQ_{av}}{dt}$$

where $R_{av}$, $B_{av}$, and $L_{av}$ represent the viscous resistance, Bernoulli’s resistance, and blood inertance, respectively. The term with Bernoulli’s resistance in Equation (1) represents the energy loss caused by flow separation when blood flows through the orifice of the aortic valve into a larger space (e.g., the ascending aorta). Such energy loss also occurs in the distal portion of artery stenosis [41]. The three parameters can be theoretically calculated based on the geometric parameters of the aortic valve and distal outflow tract as follows:

(2) $$R_{av}=\frac{8\alpha_{R}\pi \mu l}{{\left(EOA\right)}^{2}}\;,\;B_{av}=\frac{1}{2}\rho \alpha_{B}{\left(\frac{1}{EOA}-\frac{1}{A_{do}}\right)}^{2}\;,\;L_{av}=2\pi \rho \alpha_{L}\sqrt{\frac{1}{EOA}-\frac{1}{A_{do}}}$$

where μ and ρ denote the dynamic viscosity and density of blood, respectively, l is the effective length of aortic valve leaflet, and $A_{do}$ is the nominal cross-sectional area of the distal outflow tract. *EOA* denotes the effective orifice area of the aortic valve. $\alpha_{R}$, $\alpha_{B}$ and $\alpha_{L}$ are coefficients, which were set to be 0.01, 1.0, and 1.0, respectively [35]. 

The severity of aortic valve stenosis (AS) or aortic valve regurgitation (AR) was controlled by the value of *EOA* during systole or diastole, which is a major determinant of $R_{av}$, $B_{av}$, and $L_{av}$, as indicated via Equation (2). For a normal aortic valve, *EOA* was set to 4.0 cm^2^ during systole and fixed at 0 during diastole [35]. In our study, systolic *EOA* was reduced to 1.0 cm^2^ (25% of the normal value) to represent a severe AS condition, whereas diastolic *EOA* was increased to 0.3 cm^2^ to represent a severe AR condition. 

**Figure 4:**
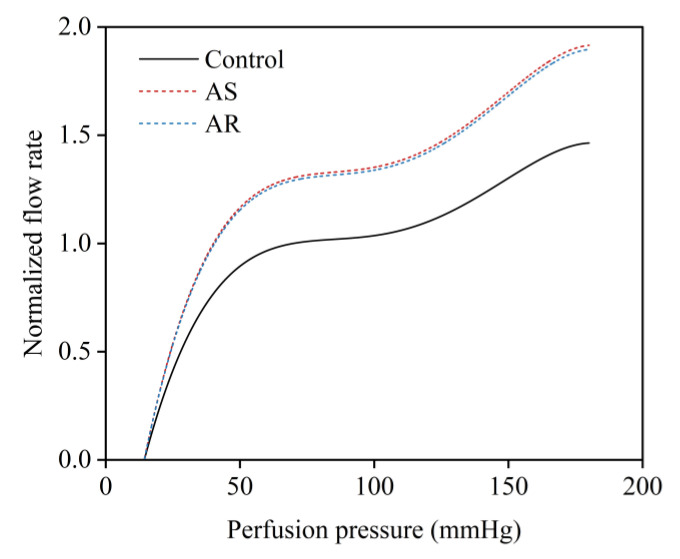
Relationships between coronary perfusion pressure and normalized mean flow rate under normal conditions and in the presence of aortic valve diseases (i.e., AS and AR).

In the presence of coronary artery stenosis, the decreased perfusion pressure and blood flow rate in the post-stenosis myocardial region will evoke the coronary flow autoregulation mechanism that acts to regulate the blood flow rate toward the normal physiological value by dilating intramyocardial microvessels [42,43]. The mechanism can be represented by a perfusion pressure–flow rate (P-Q) curve, which can be determined in in vivo studies and used as a reference in a computational model for adjusting the resistance of intramyocardial microvessels under various coronary artery stenosis conditions [21]. On the other hand, long-term presence of AS or AR will stimulate the heart to develop hypertrophic remodeling, which is usually accompanied by dilation of the coronary microcirculation that contributes to preserving sufficient blood supply to the myocardium. The resting coronary blood flow rate in a heart with AS or AR should be higher than that in a normal heart [6,7]. To incorporate the influence of aortic valve disease on resting coronary blood flow into the flow autoregulation mechanism, we shifted the P-Q curve upward according to the degree of increase in myocardial perfusion demand (estimated by the pressure–volume area of the ventricle) caused by AS or AR (see Figure 4). 

#### 2.2.5. Parameter Assignment

The parameters in the 0D models of the systemic circulation and pulmonary circulation were initially assigned based on the data reported in previous studies [34,38] and then slightly tuned using a parameter optimization algorithm (herein the Nelder–Mead method [39]) to minimize the root mean squared error between model simulations and in vivo data. For the 0D models representing the large coronary arteries and intramyocardial vessels, since they are a simplified version of a previous 0-1D multi-scale model of the coronary circulation [21], the parameter values were estimated by combining the parameter values in the 0-1D model with reference to the principle of circuit. In addition, the resistances of intramyocardial small arteries and arterioles were later modified using a linear negative feedback method to regulate coronary flow rates under various pathological conditions (i.e., coronary artery stenosis and aortic valve disease) according to the P-Q curves described in ‘Section 2.2.4’. Table 1 shows that the model-simulated systemic hemodynamic quantities and mean blood flow rates in three coronary arteries all compare reasonably with the in vivo data measured in normal subjects [44,45]. It is noted that for purpose of simplicity, we kept all the parameters in the systemic circulation model (except for those representing the aortic valve) unchanged when aortic valve disease was introduced.

### 2.3. Data Analysis

All numerical simulations were carried out under transient pulsatile flow conditions; therefore, the computed WSS (τ) in the LAD was time-varying. We integrated WSS vector or its magnitude over a cardiac cycle (T) to derive time-averaged WSS (TAWSS) and oscillatory shear index (OSI), which are two classical WSS metrics that have been widely proven to be associated with the initiation and progression of atherosclerosis [46,47,48].

(3) $$TAWSS=\frac{1}{T}\int_{0}^{T}\left|\tau \right|dt\;,\;OSI=\frac{1}{2}\left(1-\frac{\left|\int_{0}^{T}\tau dt\right|}{\int_{0}^{T}\left|\tau \right|dt}\right)$$

In addition, to facilitate quantitative comparison of hemodynamic conditions among different LADs, we further averaged TAWSS and OSI over the wall of each individual LAD to obtain space-averaged WSS metrics (herein denoted as SA-TAWSS and SA-OSI, respectively).

For each coronary artery, the simulated flow waveform at the inlet of the 3D model was further decomposed into Fourier series to analyze the waveform composition in the frequency domain as follows:

(4) $$Q\left(t\right)=Q_{0}+\sum_{n=1}^{N}Q_{n}sin\left(n\omega t+\psi_{n}\right)$$

where $Q_{0}\;$is the mean flow rate, *N* is the total number of harmonics, $Q_{n}\;$is the modulus of the *n*th harmonic, ω is the angular frequency, and $\psi_{n}\;$is the phase angle. Mathematically, the magnitudes of the moduli reflect the relative contributions of different harmonics in forming the flow waveform. 

## 3. Results

### 3.1. Changes in Cardiac Dynamics and Coronary Blood Flow Velocity Waveform Induced by Aortic Valve Disease

**Figure 5:**
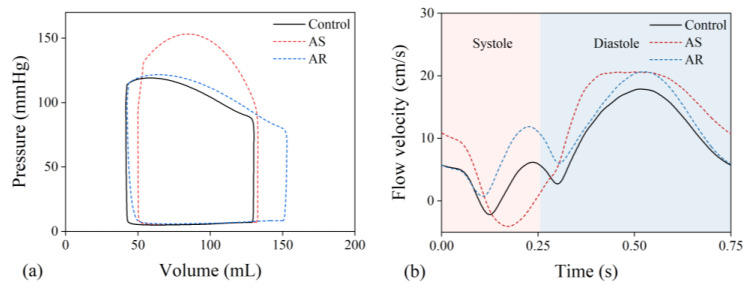
(**a**) Simulated pressure–volume loops of the left ventricle and (**b**) flow velocity waveforms in the proximal portion of a LAD under various aortic valve conditions.

The multi-scale model was employed to simulate cardiac dynamics and blood flow velocity in the LAD under the condition free of aortic valve disease (herein termed as ‘control condition’) or in the presence of severe AS or AR. Figure 5 shows the computed results for a LAD free of stenosis. It is observed that aortic valve disease leads to significant changes in both the left ventricular pressure–volume (P-V) loop and flow velocity waveform in the LAD and that the characteristics of changes are dependent strongly on the type of aortic valve disease. In the presence of severe AS, the systolic portion of the P-V loop shifted upward, and LAD flow velocity was markedly depressed in systole and augmented in diastole. In the presence of AR, the P-V loop was featured with a marked increase in stroke volume and less increase in systolic pressure, and the changes in LAD flow velocity waveform were characterized mainly by an evident increase in systolic flow velocity and a mild increase in diastolic flow velocity. Quantitative analysis of the coronary flow velocity waveforms showed that the time integration of flow velocity in systole (VTIS) was significantly lower, while that in diastole (VTID) was significantly higher in the presence of AS than under the control condition, resulting in a significantly lower S/D ratio (VTIS/VTID); in contrast, the S/D ratio was evidently higher in the presence of AR. The computed results for all six LADs are statistically summarized along with relevant in vivo measurements [11] in Table 2. From the data presented in Table 2, the differences in geometry and stenosis rate among the LADs had detectable, while relatively small influences on the characteristics of flow velocity waveform (indicated by the small standard deviations (SDs)), and all the flow velocity waveform indices (VTIS, VTID, and S/D ratio) in the presence of AS or AR differed significantly from those under the control condition. The comparison of the numerical results with in vivo measurements was good under the control condition but exhibited relatively large discrepancies for some indices (e.g., VTIS and S/D ratio) under the AS or AR condition. The reason may be complex since the pathological conditions of the patients involved in the in vivo study were diverse and our study only investigated six LADs under fixed AS or AR conditions.

### 3.2. Influences of Aortic Valve Disease on Wall Shear Stresses and Flow Patterns in the LADs

**Figure 6:**
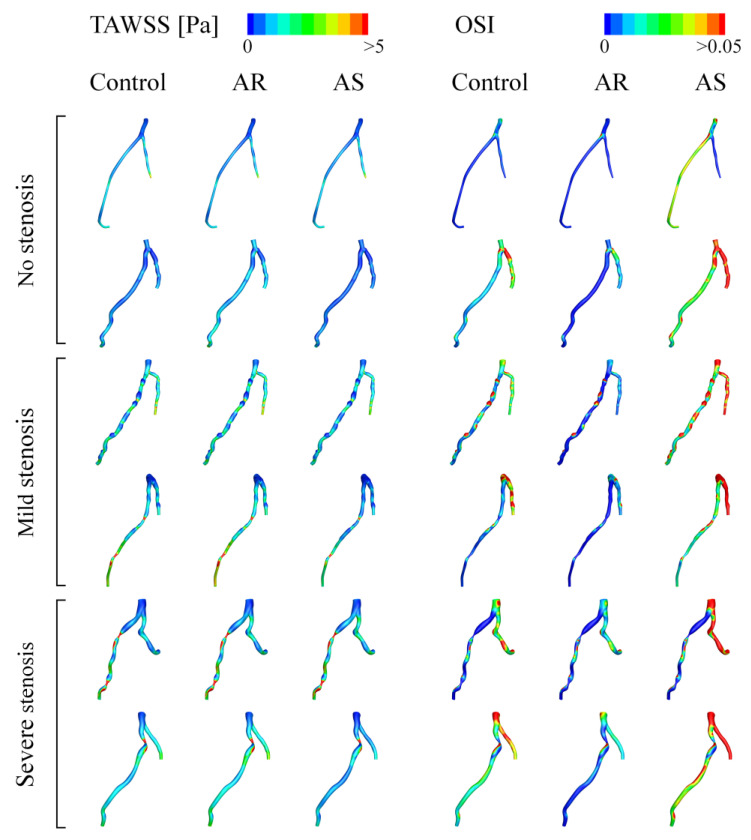
Color maps of simulated TAWSS and OSI in LADs under various aortic valve conditions.

**Figure 7:**
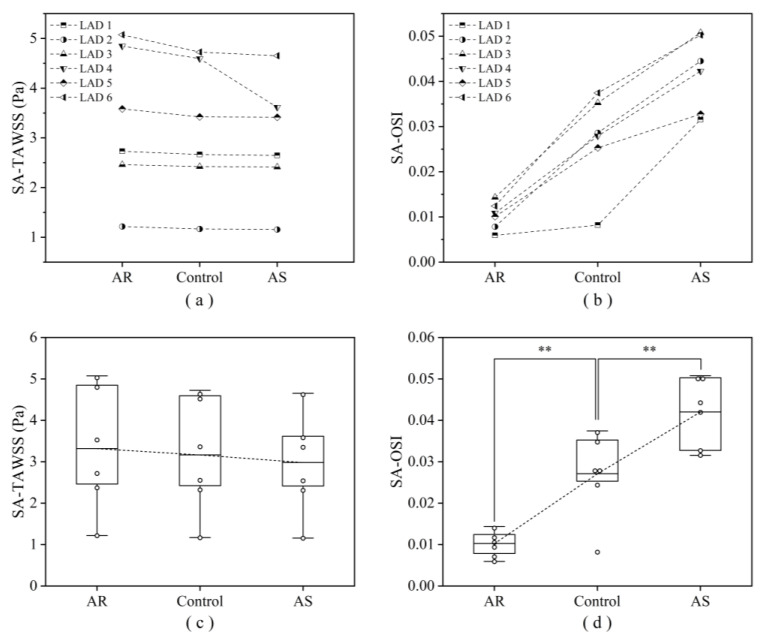
Computed SA-TAWSS (**a**) and SA-OSI (**b**) in six LADs under various aortic valve conditions, and the corresponding statistical box plots (**c**, **d**). Large box represents the lower and upper quartiles, with the line in the box denoting the mean value. ** *p* < 0.001 vs. control.

Computed hemodynamic variables in the six LADs under various aortic valve conditions were analyzed in detail in terms of the spatial distributions of TAWSS and OSI. Figure 6 shows the color maps of TAWSS and OSI in the six LADs (which are herein grouped into ‘no stenosis’, ‘mild stenosis’, and ‘severe stenosis’ groups). The color maps of TAWSS and OSI both differed evidently among LADs, indicating the dominant role of the geometry of LAD in determining the spatial distribution of WSS. Specifically, stenosis, which induces abrupt local geometric changes, is an important factor affecting the distribution of WSS, leading to the appearance of focal low or high TAWSS and high OSI in certain regions. Introducing aortic valve disease (AS or AR) did not evidently alter the global spatial distribution characteristics of TAWSS and OSI but induced considerable magnitude changes, especially in OSI. It was observed that OSI was remarkably elevated in the presence of AS. To facilitate quantitative comparison, the values of SA-TAWSS and SA-OSI (obtained by integrating TAWSS and OSI over the trunk of LAD) in all the six LADs under various cardiac valve conditions (i.e., AR, no disease (control), and AS) were plotted in Figure 7a,b, and the statistical comparisons of SA-TAWSS and SA-OSI grouped by aortic valve disease are illustrated in Figure 7c,d. As expected, SA-TAWSS and SA-OSI both exhibited considerable differences among LADs (Figure 7a,b). Statistically, SA-TAWSS was overall less affected by aortic valve disease (Figure 7c), whereas SA-OSI was markedly elevated in the presence of AS (Figure 7d). Interestingly, AR considerably reduced SA-OSI. 

**Figure 8:**
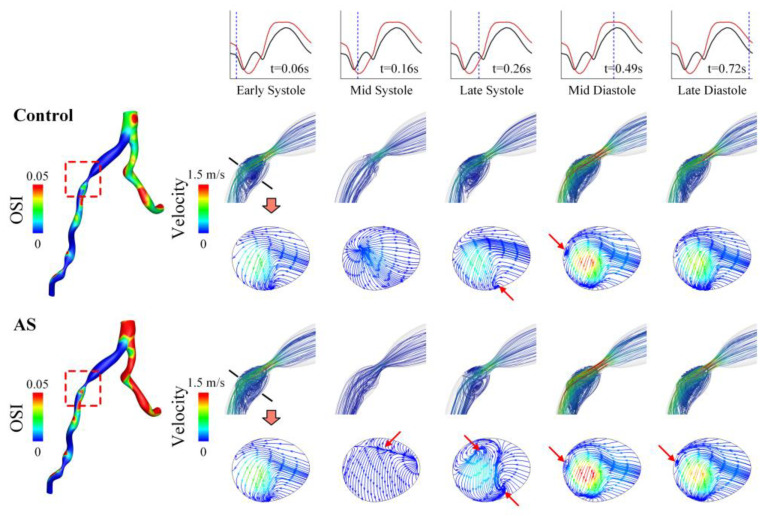
Spatial distribution of OSI (**left**) and flow streamlines (**right**) in a LAD with severe stenosis under control and AS conditions. The 3D flow streamlines in the vicinity of stenosis and 2D flow streamlines in a cross-section in the post-stenosis region are visualized for five representative time moments (marked on the flow waveforms in the upper panel) during a cardiac cycle. The red arrows indicate the regions where in-plane flow disturbance or recirculation occurs.

**Figure 9:**
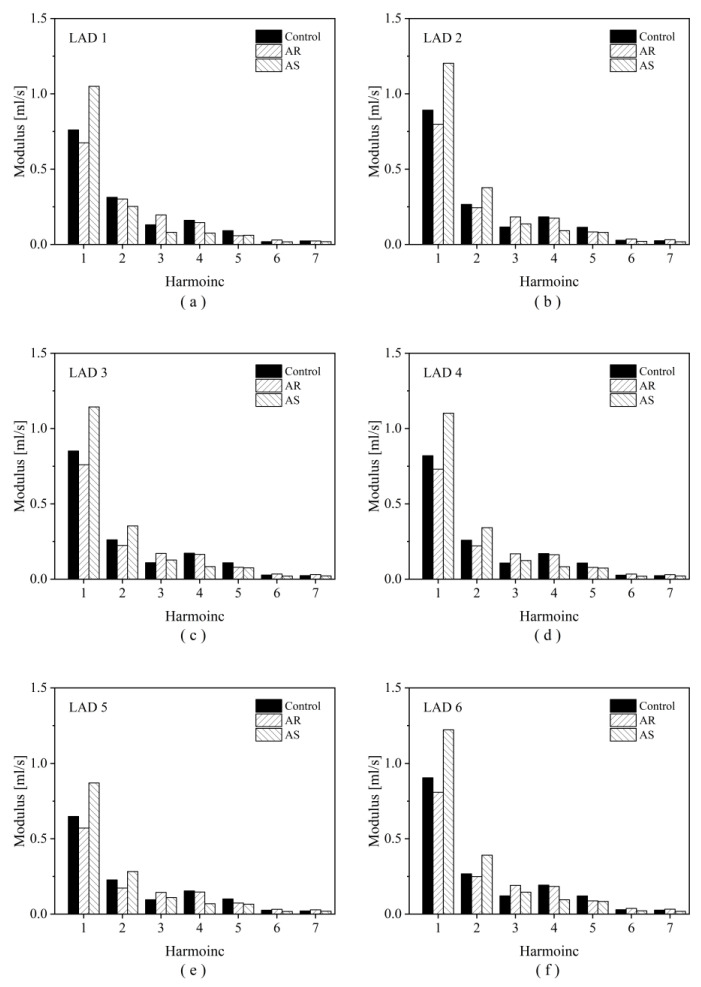
Harmonic moduli of the simulated flow waveforms in six LADs under different aortic valve conditions. Panels (**a**–**f**) display the results corresponding to LAD1 to LAD6 in sequence.

To explain why OSI increases evidently in the presence of AS, the transient blood flow patterns in a LAD with severe stenosis are illustrated in terms of 3D flow streamlines and cross-sectional 2D flow streamlines in Figure 8. In the presence of AS, flow disturbance (indicated by vortices and secondary flow) was enhanced in the post-stenosis region during systole, which might contribute to the elevation of OSI in the region. Since the geometry of the LAD was fixed, the enhancement of flow disturbance could be attributed to the increased pulsation of flow during systole (see upper panels of Figure 8). Inspired by this observation, we further analyzed the coronary flow waveforms in the frequency domain. The results show that the moduli of the low-frequency harmonics (especially harmonic 1) increased evidently following the introduction of AS (Figure 9). Therefore, the augmented magnitudes of low-frequency components in the flow waveform may represent a major mechanism by which AS elevates OSI in the LAD. On the contrary, AR reduced the moduli of the low-frequency harmonics and thereby leading to the decrease in OSI in the LAD. 

## 4. Discussion

Concurrent AVD and CAD are common in patients suffering from cardiac diseases [1,4,5]. So far, it remains unclear whether AVD would affect the risk or the progression of CAD. In this study, we developed a 0-3D multi-scale model to quantitatively investigate the influences of various AVDs on hemodynamic characteristics in coronary arteries. A major advantage of our model over the models reported in previous relevant studies [18,19,20] lies in its ability to spontaneously incorporate the hemodynamic effects of cardiac valve disease and coronary flow autoregulation mechanism into the simulation of blood flow in any large coronary artery of interest. The model was applied to quantify hemodynamic changes in six patient-specific LADs following the introduction of AS or AR. The main findings are twofold: (1) the hemodynamic environment in the LAD was deteriorated by AS, as manifested by a marked increase in OSI, but was moderately improved by AR; (2) AS-induced hemodynamic deterioration in the LAD was mediated mainly by the augmentation of the low-frequency components of coronary blood flow waveform.

High OSI indicates the intensive directional changes in WSS during a cardiac cycle, which may induce endothelial dysfunction and phenotypic changes that are associated with the initiation and development of atherosclerosis [49]. It has been demonstrated that OSI can predict the locations of plaques in LADs [48] and is independently associated with plaque erosion [50]. Given the finding of our study that AS induced a marked elevation of OSI in both normal LADs and stenosed LADs, it is reasonable to speculate that AS may not only increase the susceptibility of normal coronary arteries to future atherosclerosis but also promote the progression of existing atherosclerotic lesions. In fact, clinical studies have observed a higher incidence of CAD in patients with AS than in patients with AR (39% vs. 25% (n = 151) [51], and 57.5% vs. 44.4% (n = 76) [52]). In a sense, our findings may be taken as a new biomechanical mechanism for explaining the high prevalence of CAD in patients with AS.

The elevation of OSI in the LADs by AS is attributable to the changes in blood flow waveform (e.g., impaired systolic flow while augmented diastolic flow) as a consequence of the combined effects of altered myocardial pressure and compensatory decrease in coronary microvascular resistance [22,23,35]. Previous numerical studies have demonstrated that the flow patterns and WSS metrics in coronary arteries are sensitive to the shape and magnitude of inlet flow waveform [53,54]. A study on aneurysms presented at the internal carotid artery (ICA) revealed that the changes in blood flow waveform in the ICA during aging, which were characterized mainly by the enhancement of low-frequency components, led to an obvious increase in OSI in aneurysms [55]. In this study, the moduli of low-frequency harmonics (i.e., the first and second harmonics) of coronary artery blood flow waveform were much larger under the AS condition and smaller under the AR condition in comparison with those under the control condition (see Figure 9), which corresponded to the higher SA-OSI and lower SA-OSI in the LADs, respectively. Given the different shapes of flow waveforms in the ICA and LAD and the different anatomical features of the ICA aneurysm and LAD, the enhancement of low-frequency components may represent a general characteristic of flow waveform that contributes to the elevation of OSI in arteries. 

A major limitation of this study is that the geometric models of coronary arteries were reconstructed based on medical images taken from patients suspected of CAD rather than from those with confirmed aortic valve disease. In addition, parameters in the 0D models were assigned to reproduce the general rather than patient-specific characteristics of systemic hemodynamics. Therefore, the computed results could not fully represent the in vivo hemodynamic conditions in specific patients, only indicating how the presence of certain aortic valve disease would alter hemodynamic characteristics in coronary arteries. Nevertheless, the 0-3D model proposed in our study may be personalized to patients with concurrent AVD and CAD if sufficient clinical data required for calibrating model parameters were available. Moreover, only six LADs were studied, and the small sample size might compromise the reliability of the statistical analysis, although the between-group differences in SA-OSI were found to be statistically significant. At this point, future studies that involve a larger number of coronary arteries would be warranted in order to further confirm our findings.

## 5. Conclusions

In this study, a 0-3D multi-scale modeling method has been developed to quantify AVD-induced hemodynamic changes in six patient-specific coronary arteries. The results demonstrated that AS remarkably elevated OSI in both normal and stenosed coronary arteries, whereas AR moderately decreased OSI, which provided biomechanical evidence for explaining why the prevalence of CAD is higher in patients with AS than those with AR. In addition, it was found that the increase in OSI in the coronary artery in the presence of AS was mediated by the changes in flow waveform characterized by the augmentation of the low-frequency components. On the other hand, this study is limited by the small sample size and the lack of sufficient clinical data for building fully patient-specific models, which would be addressed by future large-scale patient-specific studies.

## Figures and Tables

**Table 1:** Comparisons of model simulations and in vivo measurements under normal conditions.

	In Vivo Measurement	Simulation
CO (L/min)	5.19 ± 0.83 [44]	4.9
SBP (mmHg)	113.0 ± 5.0 [44]	115.3
DBP (mmHg)	74.0 ± 8.0 [44]	76.5
*Q*_LAD_ (mL/s)	1.27 ± 0.56 [45]	1.25
*Q*_RCA_ (mL/s)	1.14 ± 1.28 [45]	1.13
*Q*_LCX_ (mL/s)	0.91 ± 0.41 [45]	0.89

The in vivo data are presented in form of mean ± SD (standard deviation); CO, cardiac output; SBP, systolic blood pressure; DBP, diastolic blood pressure.

**Table 2:** Comparison of characteristic indices of simulated flow velocity waveforms in six LADs with in vivo measurements under various aortic valve conditions.

	Control		AS		AR	
	MEAS [11]	SIM	MEAS [11]	SIM	MEAS [11]	SIM
VTIS (cm)	4.0 ± 1.0	3.08 ± 0.2	3.8 ± 1.4	2.06 ± 0.1	10.8 ± 3.2	6.31 ± 0.3
VTID (cm)	13.3 ± 3.6	11.74 ± 3.1	14.6 ± 3.7	15.94 ± 3.3	17.1 ± 4.5	13.63 ± 3.1
S/D ratio	0.30 ± 0.03	0.26 ± 0.04	0.26 ± 0.05	0.13 ± 0.02	0.63 ± 0.10	0.46 ± 0.05

The data obtained for the six LADs are presented in form of mean ± SD (standard deviation); MEAS, in vivo measurement; SIM, simulation.

## Data Availability

The data presented in this study are available from the corresponding author upon reasonable request. For readers developing similar models, technical support from the corresponding author is also available.

## Abbreviations

AVD	aortic valve disease
CAD	coronary artery disease
AS	aortic valve stenosis
AR	aortic valve regurgitation
LAD	left anterior descending coronary artery
WSS	wall shear stress
TAWSS	time-averaged wall shear stress
OSI	oscillatory shear index
SA-TAWSS	space-averaged time-averaged wall shear stress
SA-OSI	space-averaged oscillatory shear index
0-3D	zero-three dimensional
CCTA	coronary computed tomographic angiography
DS	diameter stenosis
UDFs	user-defined functions
EOA	the effective orifice area of the aortic valve
P-Q	pressure–flow rate
CO	cardiac output
SBP	systolic blood pressure
SDP	diastolic blood pressure
VTIS	time integration of flow velocity in systole
VTID	time integration of flow velocity in diastole
S/D ratio	VTIS/VTID
ICA	internal carotid artery
